# Strong concordance between RNA structural and single nucleotide variants identified via next generation sequencing techniques in primary pediatric leukemia and patient-derived xenograft samples

**DOI:** 10.5808/GI.2020.18.1.e6

**Published:** 2020-03-31

**Authors:** Sonali P. Barwe, Anilkumar Gopalakrisnapillai, Nitin Mahajan, Todd E. Druley, E. Anders Kolb, Erin L. Crowgey

**Affiliations:** 1 Alfred I. duPont Hospital for Children, Wilmington, DE 19803, USA; 2 Washington University School of Medicine, St. Louis, MO 63110, USA

**Keywords:** error-corrected sequencing, genomics, patient derived xenograft models, pediatric cancers, structural variants

## Abstract

Acute leukemia represents the most common pediatric malignancy comprising diverse subtypes with varying prognosis and treatment outcomes. New and targeted treatment options are warranted for this disease. Patient-derived xenograft (PDX) models are increasingly being used for preclinical testing of novel treatment modalities. A novel approach involving targeted error-corrected RNA sequencing using ArcherDX HemeV2 kit was employed to compare 25 primary pediatric acute leukemia samples and their corresponding PDX samples. A comparison of the primary samples and PDX samples revealed a high concordance between single nucleotide variants and gene fusions whereas other complex structural variants were not as consistent. The presence of gene fusions representing the major driver mutations at similar allelic frequencies in PDX samples compared to primary samples and over multiple passages confirms the utility of PDX models for preclinical drug testing. Characterization and tracking of these novel cryptic fusions and exonal variants in PDX models is critical in assessing response to potential new therapies.

## Introduction

Genomic characterization of the somatic landscape is essential for the robust clinical evaluation and classification of pediatric leukemias [1]. Somatic variants can inform both diagnosis and prognostication, as well as guide therapy decisions [2]. The development and validation of new targeted therapies for pediatric leukemias is dependent on the availability of pre-clinical models capable of recapitulation of the disease. Patient-derived orthotopic xenograft models (PDX) are routinely used in disease modeling for preclinical drug evaluation [3]. Although several studies have been conducted to understand the stability and suitability of PDX models, the majority of these efforts have focused on adult-derived leukemias and the characterization of single nucleotide variants (SNVs) [4].

Chromosomal rearrangements generating gene fusions and other structural variants (StVs) are more common in pediatric malignancies compared to adults [5]. These StVs and SNVs have demonstrated a different landscape for diagnostic, prognostic, and therapeutic value. Of note, pediatric leukemias are genomically heterogenous and require a broad spectrum of molecular biology techniques to fully characterize. Additionally, StVs are difficult to identify via short read DNA-seq approaches, and recent research has demonstrated the power and utility of identifying SNVs in RNA molecules [6].

Acute lymphoblastic leukemia (ALL) is the most common type of cancer in children and adolescents. ALL represents 20% of all cancers diagnosed in individuals with less than 20 years of age [7]. In general, survival in ALL has improved significantly over the past 40 years with more than 90% of patients now surviving. Acute myeloid leukemia (AML) is the second most common type of leukemia diagnosed in children. AML has an overall survival rate that is less than 65%. In all children with AML, and many with ALL, survival comes at the expense of intensive chemotherapy. New strategies are needed, as are preclinical models that reflect the clinical disease.

The goal of this study was to characterize complex genomic variants in pediatric leukemias and describe and monitor these variants in preclinical PDX models in comparison with the primary samples. The ability to track complex genomic lesions in primary samples and across passage in PDX lines is essential in ensuring that that the model can be used for biologic and therapeutic modeling. RNA next generation sequencing (NGS) techniques enable a sensitive and broad approach for analyzing complex genomic lesions and identifying clinically relevant novel somatic mutations associated with pediatric leukemias.

## Methods

### Patient samples and consent

All samples used in this study were procured by the Nemours Biobank following written informed consent. For majority of samples, leukemic cells were isolated from human bone marrow aspirates with the exception of NTPL-59 and NTPL-109, which were isolated from apheresis products by Ficoll density gradient centrifugation and provided to us under an Institutional Review Board approved protocol (Nemours Office of Human Subjects Protection IRB# 267207). Summary of the subject’s characteristics are presented in Table 1.

### Generation of PDX models

PDX models were generated as described previously [8] using a protocol approved by the Nemours Institutional Animal Care and Use Committee. Leukemic cells from patient samples were injected into immune-deficient NSG-B2m mice (stock no. 010636, Jackson Laboratories, Bar Harbor, ME, USA) via the tail vein. Disease progression was examined by determination of the percentage of human leukemic cells in mouse peripheral blood by flow cytometry. Mice were closely monitored for experimental endpoints such as increased leukemic burden, weight loss greater than 20% body weight, hunched back, and impaired mobility. Mice meeting end point criteria were euthanized using a method consistent with the guidelines of the American Veterinary Medical Association. Leukemic cells were isolated from the femurs and spleen post euthanasia and used for serial transplantation in a new cohort of mice. Bioauthentication and validation of PDX sample with matching primary sample was performed by subjecting the DNA samples to AmpFISTR Identifiler PCR Amplification Kit (Applied Biosystems, Foster City, CA, USA).

### Error-corrected sequencing library preparation and sequencing

To optimize detection of structural and copy number variants in RNA we prepared RNA–error-corrected sequencing libraries using the ArcherDX (Boulder, CO, USA) FusionPlex HemeV2 Kit (catalog no. AB0012) per manufacturer’s protocols. Total RNA was extracted using RNeasy Mini Kit (Qiagen, Hilden, Germany). Nucleic acid quantity and quality was then assessed using the Agilent (Santa Clara, CA, USA) TapeStation 4200 following the manufacturer’s protocol and using the High Sensitivity RNA Screen Tape (catalog no. 5067-5579). cDNA was made from 50 ng of RNA using the QIAseq kit. Each library was sequenced on the Illumina NextSeq platform (San Diego, CA, USA). The gene fusion data produced by the Archer panel was initially correlated with diagnostic fluorescence in situ hybridization data available for each primary sample.

### Bioinformatics

The data was processed via ArcherDX Analysis platform (v5.1.3), hosted in the cloud by Amazon Web Services, including fastq trimming, read deduplication, genome alignment, and variant detection and annotation. The analysis pipeline contains the following applications: ABRA [9], bamaddrg, bcftools, bedtools, blast [10], bowtie2 [11], bwa, EMBOSS, fastqc, freeBayes [12], Lofreq [13], MiXCR [14], Muscle, samtools, VEP [15], Velvet [16], HTSeq [17], complete-striped-smith-waterman-library, JBrowse [18], JQuery DataTables, Django Solo, and plot.js.

Fastq files were analyzed via fastqc for library quality, and error corrected reads (hamming distance of 2) were aligned to the genome build hg19 using BWA and bowtie2, and alignment files were processed via GATK best practices [19]. SNVs and short InDels (≤ 20 bp) were detected from the genomic alignments (forced reference mapping) by freeBayes and Lofreq, whereas large structural variants and cryptic fusions were detected via de novo assembly approaches. A minimum of three reads per unique molecular barcode (UMI) was required for the downstream process of de-duplication and error-correction [20]. Variants were filtered based on depth of error-corrected sequencing bins, minimum of 3, that supported the call. All regions in which variants called required a total read depth >100×, and a minimal base quality score (phred) of 20 was applied. The ExAC database was used to annotate common variants.

Variant allele frequencies (VAF) were calculated for SNVs based on number reads mapped to that location supporting the alternative allele versus the total number of reads mapped to that genome location. VAFs for StVs are calculated by analyzing the number of reads supporting the wild type sequence/junction, compared to the number of reads supporting the novel junction. R statistics was used for making scatter plots, specifically ggplot2 [21]. Alignment files (bam) were visualized via integrative genome browser (IGV). The fastq data is publicly available via short read archives under the following accession number (will add upon acceptance of manuscript).

## Results

### Comparison of RNA StVs and SNVs between primary and PDX AML samples

**Fig. 1.**
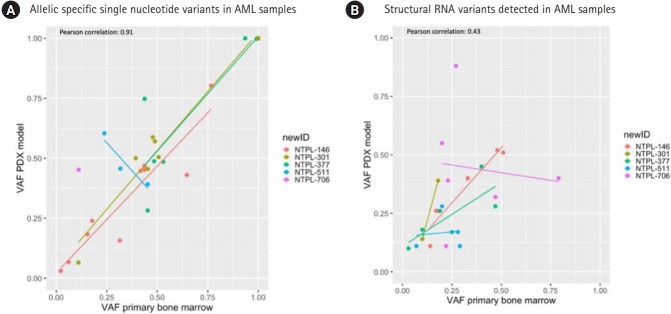
Summary of primary and xenograft RNA variants in acute myeloid leukemia (AML). (A) Allelic specific single nucleotide variants. Variant allele frequency (VAF) at time of diagnosis, x-axis is plotted versus the VAF in the xenograft model, y-axis. (B) Structural RNA variants. VAF at time of diagnosis, x-axis is plotted versus the VAF in the xenograft model, y-axis. PDX, patient-derived xenograft.

To determine the concordance of RNA variants between primary and PDX samples for pediatric AML, a targeted RNA sequencing panel approach (HemeV2; ArcherDx) was utilized. In this report, we analyzed 5 AML primary-PDX sample pairs, and in total 31 allelic specific SNVs were identified with the following distribution: 1 frameshift, 11 missense, 2 splice region and 17 untranslated region (UTR) variants (Supplementary Table 1). Five UTR variants were present at a VAF of 1 in both primary and PDX AML samples. The absolute change in VAFs between primary and PDX samples was less than 0.2 for 27 SNVs. A few variants increased in VAF in the PDX (*MYC, CDKN2A*, and *NOTCH1*), other variants reduced in VAF in PDX samples (*CCND3* and *ABL2*) (Fig. 1A, Supplementary Table 1).

VAFs for all RNA StVs including gene fusions and alternative exon usage variants were graphed between the primary and PDX AML samples and results are displayed (Fig. 1B). Four unique gene fusions (*KMT2A-MLLT1, KMT2A-MLLT3, NUP-98-NSD1*, and the reciprocal *NSD1-NUP98*) were identified in the primary AML samples and PDX samples. Additionally, 5 exon duplications/deletions were identified in *CEBPA* and *IRF4* (Supplementary Table 2).

Multiple retained introns (n=14) were identified in the 5 primary and PDX AML samples in the following genes: *ZCCHC7, ABL1, JAK2, IRF8, TAL1, CEBPG, ETV6, KMT2A, MLLT10, KLF2*, and *PRDM16* (Supplementary Table 2). The SNVs were more concordant between primary and PDX samples compared to StVs (Pearson correlation coefficient, 0.91; p = 5.12e-13 and 0.43; p = 0.036 respectively). Among the StVs, fusions were identified at similar VAFs in primary and PDX samples, whereas the alternative exon usage variants showed greater variability. Interestingly, the 2 AML samples with *KMT2A* gene rearrangements (NTPL-146 and NTPL-377) showed higher level of concordance between VAFs for StVs as well as SNVs.

### Comparison of RNA StVs and SNVs between primary and PDX T-ALL and B-ALL samples

**Fig. 2.**
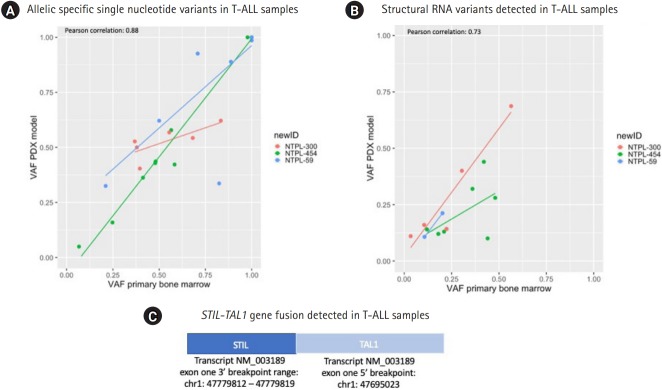
Summary of primary and xenograft RNA variants in T-cell acute lymphoblastic leukemia (T-ALL). (A) Allelic specific single nucleotide variants. Variant allele frequency (VAF) at time of diagnosis, x-axis is plotted versus the variant allele frequency in the xenograft model, y-axis. (B) Structural RNA variants. VAF at time of diagnosis, x-axis is plotted versus the VAF in the xenograft model, y-axis. (C) *STIL-TAL1* gene fusion identified in 2 of the T-ALL samples. PDX, patient-derived xenograft.

To determine the concordance of RNA variants between primary and PDX samples for pediatric ALL, samples target RNA sequencing approach was utilized. The correlation coefficients of VAF between primary and PDX T-cell ALL (T-ALL) samples identified across 3 primary and PDX T-ALL samples were similar between SNVs and StVs (Pearson correlation coefficient, 0.88; p = 6.12e-10 and 0.73; p = 0.003 respectively) (Fig. 2). In total, 25 allelic specific RNA SNVs were identified in the primary and PDX T-ALL samples: 3 frameshift, 8 missense, and 14 UTR variants. Six UTR variants had VAF = 1 in primary and PDX T-ALL samples. Three variants had absolute VAFs greater than 0.25; 1 of these SNVs (*NOTCH1* frameshift variant) reduced in VAF in PDX samples, while 2 (*CEBPA* missense variants) showed gains in PDX samples. NTPL-454 had a strong correlation between SNV VAFs in the primary and PDX models (Fig. 2A; green line), whereas NTPL-59 and NTPL-300 were not as consistent with VAFs for SNVs.

In total 14 StVs were identified in the primary and PDX models for T-ALL samples; 4 unique fusions (*STIL-TAL1, SPTAN1-ABL1*), 7 retained introns (*EIF4A, IRF8, KMT2A, NF1, SETD2*), and 3 molecules with exon duplications (*BCL11B* and *ZCCH7*). NTPL-300 was the most concordant for VAF of StVs in primary and PDX T-ALL samples (Fig. 2B). Of interest, 2 of the T-ALL samples had a *STIL-TAL* gene fusion, which was recently published as a potential driver / founder event [22].

The correlation between VAF from primary to PDX samples was analyzed for RNA StVs and SNVs in 17 B-cell ALL (B-ALL) samples. In total 114 RNA SNVs were identified in the primary and PDX B-ALL samples, and of those variants 4 were frameshift, 25 missense, 5 splice region, 2 stop gained and the rest were UTR variants (Supplementary Table 1). Twenty-two UTR variants (*RUNX1, IKZF3, CHIC2, CCND2, BCL2*) were detected at identical VAF of 1 in primary and PDX B-ALL samples. Five variants (4.4%) had VAFs greater than 0.25; *CHID1, ABL2* UTR variants showed decreased VAF, and *BCR, CCND2, NOTCH1* SNVs showed increased VAF in PDX samples.

**Fig. 3.**
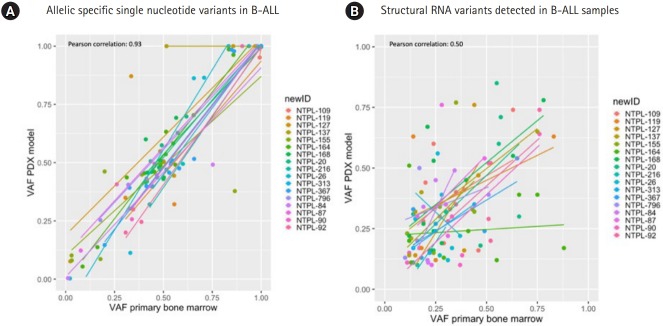
Summary of primary and xenograft RNA variants in B-cell acute lymphoblastic leukemia (B-ALL). (A) Allelic specific single nucleotide variants. Variant allele frequency (VAF) at time of diagnosis, x-axis is plotted versus the VAF in the xenograft model, y-axis. (B) Structural RNA variants. VAF at time of diagnosis, x-axis is plotted versus the VAF in the xenograft model, y-axis. PDX, patient-derived xenograft.

**Fig. 4.**
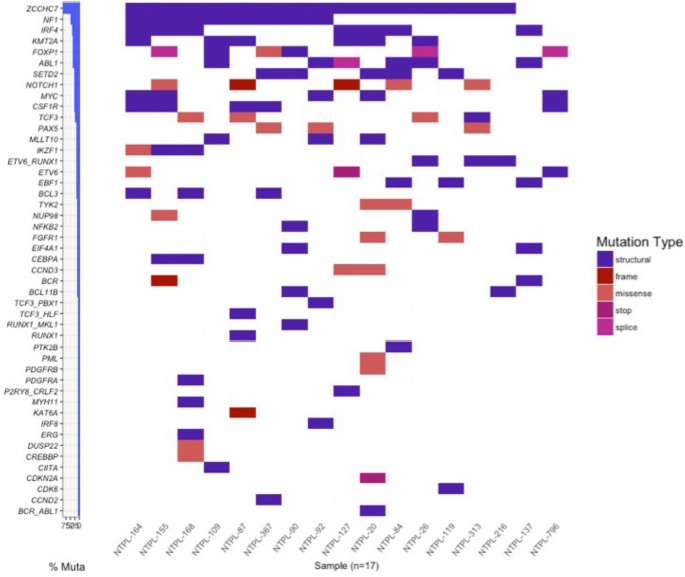
Waterfall graph for single nucleotide variants (SNVs) and structural variants (StVs) detected in B-cell acute lymphoblastic leukemia samples. Genes with either a coding SNV or StV were plotted (y-axis) per sample (x-axis). Mutations are colored based on type.

The correlation between SNV VAFs from primary to PDX B-ALL samples was higher than the correlation between StV VAFs (Pearson correlation coefficient, 0.93; p = 2.2e-16 and 0.5; p = 9.5e-8, respectively) (Fig. 3A, B). Eight of 17 samples possessed a gene fusion (*BCR-ABL1, ETV6-RUNX1, P2RY8-CRLF2, RUNX1-MKL1, TCF3-HLF, TCF3-PBX1*). The VAFs for StVs, especially the alternate exon usage variants, were more variable in these samples, similar to AML samples. Interestingly, 15 out of 18 of the B-ALL samples had a retained intron in *ZCCHC7* involving intron 2, which was persistent in PDX samples (Fig. 3B). Two AML and 1 T-ALL sample also showed a similar retained intron variant (Supplementary Table 2). *ZCCHC7* intron 2 has been mapped to hotspot for breakpoints in B-ALL [23]. *ZCCHC7* topped the waterfall graph used to analyze and visualize the most commonly altered genes in B-ALL samples (Fig. 4).

## Discussion

**Fig. 5.**
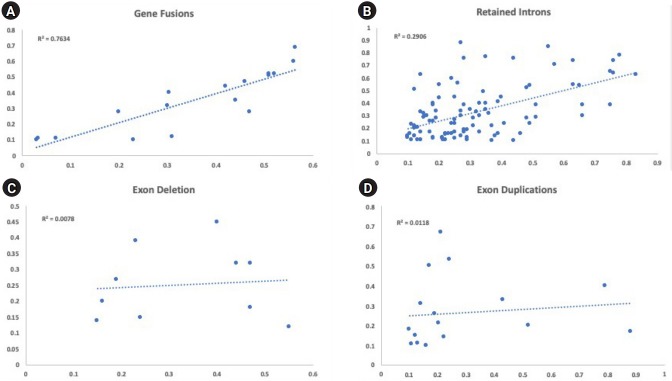
Comparison of variant allele frequencies of structural variants between primary bone marrow samples (x-axis) and matched xenograft sample (y-axis). (A) The variant allele frequencies for all gene fusions were plotted between the primary and xenograft model (R^2^ = 0.7634). (B) The variant allele frequencies for all retained introns were plotted between the primary and xenograft model (R^2^ = 0.2906). (C) The variant allele frequencies for all exon deletion were plotted between the primary and xenograft model (R^2^ = 0.0078). (D) The variant allele frequencies for all exon duplications were plotted between the primary and xenograft model (R^2^ = 0.0118).

Sequencing of primary acute leukemia patient samples and matching PDX samples showed concordance between the detected variants and their allelic frequencies for the majority of variants tested. The percentage of all variants with absolute delta VAFs <0.2 was 86.7%. This percentage was higher in SNVs (93.6%) compared to StVs (79.6%) across all primary and PDX samples analyzed. Among the different categories of StVs, the allelic frequencies of fusion genes, which are considered to be driver mutations, matched most consistently between the primary and PDX samples (Fig. 5). Our data validate this novel sequencing approach for detection and tracking of diverse variants in primary leukemic samples and corresponding PDX lines.

We identified several SNVs, but no StVs, with sustained VAF = 1 in primary and PDX samples across all leukemia subtypes. These SNVs in genes *ABL1, BCL2, CCND2, CHIC2, IKZF3, RUNX1*, and *MECOM*, likely represent the germline mutations. Several germline variants, including UTR variants have been shown to be associated with disposition to hematological malignancies [24]. Future characterization of these variants will determine the relevance of these germline UTR variants.

Retained intron variants were detected in all samples except NTPL-59. Retention of introns serves as another mode of regulation of gene expression [25]. Alternative splicing of multi-exon genes in patients with AML compared to normal CD34+ cells has been observed [26]. Such alternative exon usage variants were associated with oncogene expression and drug resistance [27]. Further work is required to understand the biological and clinical significance of alternative exon usage variants.

As we have shown previously, error-correction via the introduction of a nucleic acid-specific UMI allows the removal of NGS errors, retaining only true mutations and significantly improving the sensitivity of NGS [28-30]. In this study, we paired the error-correction strategy with anchored-multiplexed PCR (AMP) chemistry for the quantitative detection of complex structural RNA variants. Recently Benayed et al. [31] published an RNA sequencing approach similar to the one outlined in this manuscript (MSK targeted RNA panel using ArcherDx), and demonstrated that their MSK-IMPACT DNA panel missed cancer-related and targetable mutations in greater than 15% of lung cancer patients. They leveraged ArcherDx FusionPlex technology (identical to our approach) to identify these cases. Additionally, several recent studies have demonstrated the use of AMP technology (ArcherDx) for identifying rare and complex structural variants in pediatric cancers [32,33].

Taken together, advanced sequencing techniques are required to accurately detect and annotate complex StVs that are commonly associated with pediatric leukemias. Such complex variants, including StVs, are not detectable using DNA and short read sequencing technology such as Illumina sequencing platform. Additionally, the RNA molecules that are generated from these complex genomic rearrangements can be difficult to capture. Using an RNA sequencing approach with AMP technology and short read sequencing platform described in this study, pediatric PDX models could be appropriately characterized and validated for concordance of somatic mutations with respect to primary samples. Such analysis is not feasible using standard DNA sequencing techniques. This is one of the first reports to describe pediatric PDX samples using an RNA sequencing approach.

## Figures and Tables

**Table 1.** Summary of leukemic samples utilized

Patient characteristic	AML	ALL
No.	5	20
Age (yr), median (range)	10 (1.5-14)	5.5 (1-16)
Sex		
Male	40	55
Female	60	45
Race		
Caucasian	60	35
African American	0	25
Hispanic	20	20
Samples collected at diagnosis	80	95
Cytogenetically normal (by karyotype analysis)	0	55
Bone marrow origin	100	90
Peripheral blood origin	0	10
Average leukemic blast percentage	76	78

Values are presented as percentage unless otherwise indicated.

AML, acute myeloid leukemia; ALL, acute lymphoblastic leukemia.

## Supplementary Materials

Supplementary Table 1. Single nucleotide variants detected per subject.

Supplementary Table 2. Structural variants detected per subject.
