# Supplementary material for: Strong concordance between RNA structural and single nucleotide variants identified via next generation sequencing techniques in primary pediatric leukemia and patient-derived xenograft samples
Source: Genomics Inform. 2020 Mar 31;18(1):e6. doi: 10.5808/GI.2020.18.1.e6 (PMC7120351; doi:10.5808/GI.2020.18.1.e6)
Supplement: Supplementary Table 1. — Single nucleotide variants detected per subject. [file gi-2020-18-1-e6-suppl1.pdf]

ID	Type	Gene ID Patient Sample	Patient Sample Breakpoint	Exons	AF Patient	AF Xenograft	delta_AF	Type
NTPL-20	B-ALL	BCR ,Üí ABL1	chr22:23524426,chr9:133729451	Exon 1 BCR : Exon 2 ABL1	56%	60%	4%	Fusion
NTPL-20	B-ALL	ZCCHC7	chr9:37304058,chr9:37304185	Intron 3: Exon 4	57%	71%	14%	Retained intron
NTPL-20	B-ALL	SETD2	chr3:47205344,chr3:47168177|<wbr>chr3:47168154,chr3:47168153	Exon1: Intron 1: Exon 2: Exon 3	14%	11%	-3%	Retained intron
NTPL-20	B-ALL	IRF4	chr6:391809,chr6:394821	Exon 1: Exon 3	47%	18%	-29%	Exon Deletion
NTPL-20	B-ALL	KMT2A	chr11:118362033,chr11:118362034	Exon 14: Intron 14	66%	30%	-36%	Retained intron
NTPL-20	B-ALL	ZCCHC7	chr9:37187568,chr9:37302185	Exon 3: Intron 2	24%	16%	-8%	Retained intron
NTPL-20	B-ALL	MYC	chr8:128748656,chr8:128748348	Exon 1: Exon 1	14%	31%	17%	Exon Duplication
NTPL-20	B-ALL	MLLT10	chr10:21957852,chr10:21957853	Exon 9: Intron 8	55%	85%	30%	Retained intron
NTPL-26	B-ALL	ETV6 ,Üí RUNX1	chr12:12006495,chr21:36265259	Exon 4 ETV6: Exon 3 RUNX1	31%	12%	-19%	Fusion
NTPL-26	B-ALL	ABL1	chr9:133747513,chr9:133747516	Exon 5: Intro 4	37%	22%	-15%	Retained intron
NTPL-26	B-ALL	NFKB2	chr10:104161916,chr10:104161917	Exon 22: Intron 22	28%	39%	11%	Retained intron
NTPL-26	B-ALL	KMT2A	chr11:118307659,chr11:118309739|<wbr>chr11:118309837,chr11:118339490	Exon 2: Intron 1: Exon 1	15%	29%	14%	Retained intron
NTPL-26	B-ALL	ZCCHC7	chr9:37188456,chr9:37302186	Exon 3: Intron 2	33%	17%	-16%	Retained intron
NTPL-26	B-ALL	NUP98	chr11:3781718,chr11:3781715	Exon 10: Intron 10	26%	56%	30%	Retained intron
NTPL-84	B-ALL	SETD2	chr3:47205344,chr3:47168177|<wbr>chr3:47168154,chr3:47168153	Exon 1: Intron 1: Exon 2	21.30%	11%	-10%	Retained intron
NTPL-84	B-ALL	EBF1	chr5:158134987,chr5:158134986	Exon 15: Intron 15	34.30%	49%	15%	Retained intron
NTPL-84	B-ALL	IRF4	chr6:397252,chr6:401424	Exon 7: Exon 5	16%	20%	4%	Exon Deletion
NTPL-84	B-ALL	PTK2B	chr8:27251626,chr8:27255059|<wbr>chr8:27255064,chr8:27255065	Exon 3: Intron 2	25%	45%	20%	Retained intron
NTPL-84	B-ALL	ZCCHC7	chr9:37187566,chr9:37188334|<wbr>chr9:37188456,chr9:37302186	Exon 3: Intron 2	19%	34%	15%	Retained intron
NTPL-84	B-ALL	ABL1	chr9:133726934,chr9:133729451	Exon 2: Intron 1	21%	12%	-9%	Retained intron
NTPL-87	B-ALL	TCF3 ,Üí HLF	chr19:1619318,chr17:53398022	Exon 14 TCF3: Exon 4 HLF	23%	10%	-13%	Fusion
NTPL-87	B-ALL	ZCCHC7	chr9:37144995,chr9:37302186	Exon 3: Intron 2	30%	35%	5%	Retained intron
NTPL-87	B-ALL	RUNX1	chr21:36206707,chr21:36206706	Exon 4: Intron 4	31%	22%	-9%	Retained intron
NTPL-87	B-ALL	KMT2A	chr11:118362033,chr11:118362034	Exon 14: Intron 14	49%	54%	5%	Retained intron
NTPL-87	B-ALL	CSF1R	chr5:149446589,chr5:149441412	Exon 11: Intron 10	28%	76%	48%	Retained intron
NTPL-87	B-ALL	NF1	chr17:29637630,chr17:29652838	Exon 36: Intron 35	16%	30%	14%	Retained intron
NTPL-90	B-ALL	RUNX1 ,Üí MKL1	chr21:36421139,chr22:40831558	Exon 2 RUNX1: Exon 2 MKL1	51%	52%	1%	Fusion
NTPL-90	B-ALL	ZCCHC7	chr9:37284323,chr9:37302185	Exon 3: Intron 2	76%	64%	-12%	Retained intron
NTPL-90	B-ALL	EIF4A1	chr17:7477576,chr17:7477578	Exon 2: Intron 1	38%	14%	-24%	Retained intron
NTPL-90	B-ALL	ZCCHC7	chr9:37187568,chr9:37302185	Exon 3: Intron 2	17%	17%	0%	Retained intron
NTPL-90	B-ALL	SETD2	chr3:47205344,chr3:47168177|<wbr>chr3:47168154,chr3:47168153	Exon 1: Intron 1: Exon 2	13%	11%	-2%	Retained intron
NTPL-90	B-ALL	BCL11B	chr14:99723808,chr14:99724176	Exon 2: Exon 2	12%	15%	3%	Exon Duplication
NTPL-90	B-ALL	FOXP1	chr3:71348971,chr3:71348968	Exon 5: Intron 5	33%	30%	-3%	Retained intron
NTPL-90	B-ALL	NFKB2	chr10:104161916,chr10:104161917	Exon 22: Intron 22	37%	10%	-27%	Retained intron
NTPL-90	B-ALL	NF1	chr17:29637630,chr17:29652838	Exon 36: Intron 35	11%	11%	0%	Retained intron
NTPL-127	B-ALL	P2RY8 ,Üí CRLF2	chrX:1655814,chrX:1331530	Exon 1 P2RY8: Exon 1 CRLF2	46%	47%	1%	Fusion
NTPL-127	B-ALL	ZCCHC7	chr9:37284323,chr9:37302185	Exon 3: Intron 2	44%	76%	32%	Retained intron
NTPL-127	B-ALL	KMT2A	chr11:118367082,chr11:118367084	Exon 20: Intron 20	39%	16%	-23%	Retained intron
NTPL-127	B-ALL	IRF4	chr6:397252,chr6:401424	Exon 7: Exon 5	15%	14%	-1%	Exon Deletion
NTPL-137	B-ALL	EIF4A1	chr17:7477576,chr17:7477578	Exon 2: Intron 1	75%	65%	-10%	Retained intron
NTPL-137	B-ALL	EBF1	chr5:158139978,chr5:158139975	Exon 13: Intron 13	13%	21%	8%	Retained intron
NTPL-137	B-ALL	BCR	chr22:23637342,chr22:23637343	Exon 16: Intron 16	18%	40%	22%	Retained intron
NTPL-137	B-ALL	ABL1	chr9:133726934,chr9:133729451	Exon 2: Intron 2	15%	32%	17%	Retained intron
NTPL-137	B-ALL	IRF4	chr6:397110,chr6:397108	Exon 5: Intron 4	12%	14%	2%	Retained intron
NTPL-146	AML	KMT2A ,Üí MLLT1	chr11:118353210,chr19:6270770	Exon 8 KMT2A: Exon 2 MLLT1	51%	51%	0%	Fusion
NTPL-146	AML	ZCCHC7	chr9:37187568,chr9:37302185	Exon 3: Intron 2	33%	40%	7%	Retained intron
NTPL-146	AML	ABL1	chr9:133710912,chr9:133729451	Exon 2: Intron 1	17%	26%	9%	Retained intron
NTPL-146	AML	JAK2	chr9:5079263,chr9:5080229	Exon 17: Intron 16	14%	11%	-3%	Retained intron
NTPL-146	AML	IRF8	chr16:85936620,chr16:85936621	Exon 2: Intron 1	18%	26%	8%	Retained intron
NTPL-146	AML	TAL1	chr1:47697421,chr1:47695023	Exon 2: Intron 1	48%	52%	4%	Retained intron
NTPL-301	AML	CEBPG	chr19:33865517,chr19:33865430	Exon 1: Intron 1	10%	14%	4%	Retained intron
NTPL-301	AML	ETV6	chr12:11905513,chr12:11979336	Exon 2: Intron 2	18%	39%	21%	Retained intron
NTPL-377	AML	KMT2A ,Üí MLLT3	chr11:118353210,chr9:20365742	Exon 8 KMT2A : Exon 6 MLLT3	47%	28%	-19%	Fusion
NTPL-377	AML	JAK2	chr9:5079263,chr9:5080229	Exon 17: Intron 16	25%	17%	-8%	Retained intron
NTPL-377	AML	KMT2A	chr11:118362033,chr11:118362034	Exon 14: Intron 13	40%	45%	5%	Retained intron
NTPL-377	AML	CEBPA	chr19:33793032,chr19:33792760	Exon 1: Exon 1	19%	26%	7%	Exon Duplication
NTPL-377	AML	KMT2A ,Üí MLLT3	chr11:118352807,chr9:20365742	Exon 7 KMT2A : Exon 6 MLLT3	3%	10%	7%	Fusion
NTPL-377	AML	CEBPA	chr19:33793286,chr19:33793107	Exon 1: Exon 1	10%	18%	8%	Exon Duplication
NTPL-454	T-ALL	EIF4A1	chr17:7477576,chr17:7477578	Exon 2: Intron 1	48%	28%	-20%	Retained intron
NTPL-454	T-ALL	KMT2A	chr11:118367082,chr11:118367084	Exon 20: Intron 20	44%	10%	-34%	Retained intron
NTPL-454	T-ALL	STIL ,Üí TAL1	chr1:47779708,chr1:47695023	Exon 1 STIL: Exon 2 TAL1	42%	44%	2%	Fusion
NTPL-454	T-ALL	KMT2A	chr11:118309837,chr11:118339490	Exon 2: Intron 1	18%	12%	-6%	Retained intron
NTPL-454	T-ALL	NF1	chr17:29637630,chr17:29652838	Exon 36: Intron 35	36%	32%	-4%	Retained intron
NTPL-454	T-ALL	IRF8	chr16:85942779,chr16:85943090	Exon 3: Intron 3	21%	13%	-8%	Retained intron
NTPL-454	T-ALL	NF1	chr17:29637630,chr17:29641323|<wbr>chr17:29641411,chr17:29652838	Exon 36: Intron 35	12%	14%	2%	Retained intron
NTPL-511	AML	NUP98 ,Üí NSD1	chr11:3765739,chr5:176662822	Exon 12 NUP98: Exon 6 NSD1	20%	28%	8%	Fusion
NTPL-511	AML	ABL1	chr9:133726934,chr9:133729451	Exon 2: Intron 1	28%	17%	-11%	Retained intron
NTPL-511	AML	MLLT10	chr10:21846753,chr10:21875223	Exon 3: Intron 2	29%	11%	-18%	Retained intron
NTPL-511	AML	NSD1 ,Üí NUP98	chr5:176639196,chr11:3756554	Exon 5 NSD1: Exon 13 NUP98	7%	11%	4%	Fusion
NTPL-706	AML	ETV6	chr12:11905513,chr12:11979336	Exon 2: Intron 2	20%	55%	35%	Retained intron
NTPL-706	AML	CEBPA	chr19:33793042,chr19:33792763	Exon 1: Exon 1	79%	40%	-39%	Exon Duplication
NTPL-706	AML	IRF4	chr6:391809,chr6:394821	Exon 3: Exon 1	47%	32%	-15%	Exon Deletion
NTPL-706	AML	KLF2	chr19:16435809,chr19:16435810|<wbr>chr19:16435814,chr19:16437672	Exon 3: Intron 1: Exon 1	22%	11%	-11%	Retained intron
NTPL-706	AML	IRF4	chr6:397252,chr6:401424	Exon 7: Exon 5	23%	39%	16%	Exon Deletion
NTPL-706	AML	PRDM16	chr1:3013299,chr1:3102693	Exon 2: Intron 1	27%	88%	61%	Retained intron
NTPL-796	B-ALL	ETV6	chr12:11905513,chr12:11978578	Exon 2: Intron 1	10%	13%	3%	Retained intron
NTPL-796	B-ALL	MYC	chr8:128748548,chr8:128748729	Exon 1: Exon 1	17%	50%	33%	Exon Duplication
NTPL-796	B-ALL	CSF1R	chr5:149446589,chr5:149441412	Exon 11: Intron 10	51%	39%	-12%	Retained intron
NTPL-92	B-ALL	TCF3 ,Üí PBX1	chr19:1619110,chr1:164761731	Exon 15 TCF3: Exon 3 PBX1	52%	52%	0%	Fusion
NTPL-92	B-ALL	ZCCHC7	chr9:37284323,chr9:37302185	Exon 3: Intron 2	51%	29%	-22%	Retained intron
NTPL-92	B-ALL	ZCCHC7	chr9:37187568,chr9:37302185	Exon 3: Intron 2	31%	28%	-3%	Retained intron
NTPL-92	B-ALL	MYC	chr8:128748656,chr8:128748348	Exon 1: Exon 1	52%	20%	-32%	Exon Duplication
NTPL-92	B-ALL	MLLT10	chr10:21957852,chr10:21957853	Exon 9: Intron 8	76%	74%	-2%	Retained intron
NTPL-92	B-ALL	NF1	chr17:29637630,chr17:29652838	Exon 36: Intron 35	35%	35%	0%	Retained intron
NTPL-92	B-ALL	ABL1	chr9:133729453,chr9:133729451	Exon 2: Intron 1	35%	40%	5%	Retained intron
NTPL-92	B-ALL	IRF8	chr16:85936462,chr16:85936614|<wbr>chr16:85936620,chr16:85936621	Exon 2: Intron 1	39%	41%	2%	Retained intron
NTPL-109	B-ALL	ZCCHC7	chr9:37187568,chr9:37302185	Exon 3: Intron 2	29%	19%	-10%	Retained intron
NTPL-109	B-ALL	KMT2A	chr11:118367082,chr11:118367084	Exon 20: Intron 20	20%	44%	24%	Retained intron
NTPL-109	B-ALL	CIITA	chr16:10992859,chr16:10992860	Exon 5: Intron 5	27%	30%	3%	Retained intron
NTPL-109	B-ALL	NF1	chr17:29637630,chr17:29652838	Exon 36: Intron 35	46%	16%	-30%	Retained intron
NTPL-109	B-ALL	KMT2A	chr11:118362033,chr11:118362034	Exon 14: Intron 14	24%	60%	36%	Retained intron
NTPL-109	B-ALL	FOXP1	chr3:71348971,chr3:71348968	Exon 5: Intron 5	19%	28%	9%	Retained intron
NTPL-109	B-ALL	MLLT10	chr10:21957852,chr10:21957853	Exon 9: Intron 8	63%	74%	11%	Retained intron
NTPL-109	B-ALL	ABL1	chr9:133710216,chr9:133729447|<wbr>chr9:133729453,chr9:133729451	Exon 2: Intron 1	32%	33%	1%	Retained intron
NTPL-119	B-ALL	ZCCHC7	chr9:37284323,chr9:37302185	Exon 3: Intron 2	83%	63%	-20%	Retained intron
NTPL-119	B-ALL	SETD2	chr3:47205344,chr3:47139571|<wbr>chr3:47139445,chr3:47139444	Exon 1: Exon 9: Intron 9	18%	17%	-1%	Exon Deletions and Retained Introns
NTPL-119	B-ALL	CDK6	chr7:92300740,chr7:92300738	Exon 5: Intron 5	25%	12%	-13%	Retained intron
NTPL-119	B-ALL	EBF1	chr5:158134987,chr5:158134986	Exon 15: Intron 15	14%	63%	49%	Retained intron
NTPL-59	T-ALL	BCL11B	chr14:99723808,chr14:99724176	Exon 2: Exon 2	20%	21%	1%	Exon Duplication
NTPL-59	T-ALL	ZCCHC7	chr9:37126939,chr9:37126309	Exon 2: Exon 2	11%	11%	0%	Exon Duplication
NTPL-300	T-ALL	BCL11B	chr14:99723808,chr14:99724176	Exon 2: Exon 2	22%	14%	-8%	Exon Duplication
NTPL-300	T-ALL	SPTAN1 ,Üí ABL1	chr9:131329256,chr9:133738150	Exon 2 SPTAN1 : Exon 4 ABL1	56%	69%	12%	Fusion
NTPL-300	T-ALL	STIL ,Üí TAL1	chr1:47779708,chr1:47695023	Exon 1 STIL : Exon 1 TAL1	30%	40%	10%	Fusion
NTPL-300	T-ALL	SETD2	chr3:47205344,chr3:47168177	Exon 1 : Intron 1	11%	16%	6%	Retained intron
NTPL-300	T-ALL	STIL ,Üí TAL1	chr1:47779708,chr1:47689770	Exon 1 STIL : Exon 3 TAL1	3%	11%	8%	Fusion
NTPL-155	B-ALL	IKZF1	chr7:50367353,chr7:50467616	Exon 3: Exon 8	44%	32%	-12%	Exon Deletions
NTPL-155	B-ALL	IRF4	chr6:397252,chr6:401424	Exon 5: Exon 7	24%	15%	-9%	Exon Deletion
NTPL-155	B-ALL	ZCCHC7	chr9:37144995,chr9:37302186	Exon 3: Intron 2	11%	23%	12%	Retained intron
NTPL-155	B-ALL	CSF1R	chr5:149446589,chr5:149441412	Exon 12: Intron 11	35%	77%	42%	Retained intron
NTPL-155	B-ALL	CEBPA	chr19:33793035,chr19:33792763	Exon 1: Exon 1	43%	33%	-10%	Exon Duplication
NTPL-155	B-ALL	MYC	chr8:128748656,chr8:128748348	Exon 1: Exon 1	16%	10%	-6%	Exon Duplication
NTPL-155	B-ALL	NF1	chr17:29637630,chr17:29652838	Exon 36: Intron 35	27%	34%	7%	Retained intron
NTPL-164	B-ALL	MYC	chr8:128748656,chr8:128748348	Exon 1: Exon 1	88%	17%	-71%	Exon Duplication
NTPL-164	B-ALL	ZCCHC7	chr9:37147442,chr9:37302185	Exon 3: Intron 2	75%	39%	-36%	Retained intron
NTPL-164	B-ALL	IRF4	chr6:391809,chr6:394821	Exon 1: Exon 3	55%	12%	-43%	Exon Deletion
NTPL-164	B-ALL	ZCCHC7	chr9:37187568,chr9:37302185	Exon 3: Intron 2	12%	20%	8%	Retained intron
NTPL-164	B-ALL	KMT2A	chr11:118362033,chr11:118362034	Exon 14: Intron 14	66%	39%	-27%	Retained intron
NTPL-164	B-ALL	BCL3	chr19:45253362,chr19:45254484	Exon 2: Intron 1	41%	24%	-17%	Retained intron
NTPL-164	B-ALL	CSF1R	chr5:149446589,chr5:149441412	Exon 12: Intron 11	12%	51%	39%	Retained intron
NTPL-164	B-ALL	NF1	chr17:29637630,chr17:29652838	Exon 36: Intron 35	22%	16%	-6%	Retained intron
NTPL-164	B-ALL	MYC	chr8:128748662,chr8:128748315	Exon 1: Exon1	13%	11%	-2%	Exon Duplication
NTPL-164	B-ALL	NF1	chr17:29637630,chr17:29641323|<wbr>chr17:29641411,chr17:29652838	Exon 36: Intron 35	29%	13%	-16%	Retained intron
NTPL-168	B-ALL	ERG	chr21:39764564,chr21:39764366	Exon 8: Intron 7	63%	55%	-8%	Retained intron
NTPL-168	B-ALL	IKZF1	chr7:50367353,chr7:50467616	Exon 8: Exon 3	40%	45%	5%	Exon Deletions
NTPL-168	B-ALL	ZCCHC7	chr9:37187568,chr9:37302185	Exon 3: Intron 2	12%	22%	10%	Retained intron
NTPL-168	B-ALL	IRF4	chr6:397252,chr6:401424	Exon 5: Exon 7	19%	27%	8%	Exon Deletion

NTPL-168	B-ALL	PDGFRA	chr4:55139703,chr4:55139704	Exon 10: Intron 9	24%	24%	0%	Retained intron
NTPL-168	B-ALL	NF1	chr17:29637630,chr17:29652838	Exon 36: Intron 35	23%	16%	-7%	Retained intron
NTPL-168	B-ALL	BCL3	chr19:45253362,chr19:45254484	Exon 2: Intron 1	28%	19%	-9%	Retained intron
NTPL-168	B-ALL	CEBPA	chr19:33792461,chr19:33792515	Exon 1: Exon 1	24%	53%	29%	Exon Duplication
NTPL-168	B-ALL	MYH11	chr16:15853081,chr16:15841653	Exon 19: Intron 18	78%	78%	0%	Retained intron
NTPL-168	B-ALL	CEBPA	chr19:33792461,chr19:33792521	Exon 1: Exon1	21%	67%	46%	Exon Duplication
NTPL-216	B-ALL	BCL11B	chr14:99723808,chr14:99724176	Exon 2: Exon 2	16%	10%	-6%	Exon Duplication
NTPL-216	B-ALL	ZCCHC7	chr9:37187568,chr9:37302185	Exon 3: Intron 2	27%	14%	-13%	Retained intron
NTPL-216	B-ALL	ETV6 ,Üí RUNX1	chr12:12022903,chr21:36265260	Exon 5 ETV6: Exon 3 RUNX1	30%	32%	2%	Fusion
NTPL-313	B-ALL	ZCCHC7	chr9:37187568,chr9:37302185	Exon 3: Intron 2	14%	17%	3%	Retained intron
NTPL-313	B-ALL	ETV6 ,Üí RUNX1	chr12:12022903,chr21:36265260	Exon 5 ETV6: Exon 3 RUNX1 : Exon 3 ETV6	44%	35%	-9%	Fusion
NTPL-313	B-ALL	TCF3	chr19:1621132, chr19:1621131	Exon 10: Intron 10	25%	24%	-1%	Retained intron
NTPL-367	B-ALL	SETD2	chr3:47205344,chr3:47168177|<wbr>chr3:47168154,chr3:47168153	Exon 1: Intron 1: Exon 2	18%	13%	-5%	Retained intron
NTPL-367	B-ALL	BCL3	chr19:45253362,chr19:45254484	Exon 2: Intron 1	49%	24%	-25%	Retained intron
NTPL-367	B-ALL	ZCCHC7	chr9:37147442,chr9:37302185	Exon 3: Intron 2	22%	15%	-7%	Retained intron
NTPL-367	B-ALL	CSF1R	chr5:149446589,chr5:149441412	Exon 12: Intron 11	65%	54%	-11%	Retained intron
NTPL-367	B-ALL	CCND2	chr12:4399615,chr12:4409026	Exon 5: Intron 4	14%	33%	19%	Retained intron
NTPL-367	B-ALL	NF1	chr17:29637630,chr17:29652838	Exon 36: Intron 35	25%	27%	2%	Retained intron
