# Supplementary material for: Strong concordance between RNA structural and single nucleotide variants identified via next generation sequencing techniques in primary pediatric leukemia and patient-derived xenograft samples
Source: Genomics Inform. 2020 Mar 31;18(1):e6. doi: 10.5808/GI.2020.18.1.e6 (PMC7120351; doi:10.5808/GI.2020.18.1.e6)
Supplement: Supplementary Table 2. — Structural variants detected per subject. [file gi-2020-18-1-e6-suppl2.pdf]

Type	Sample	AF_primary	AF_xen	delta	Gene	consequence
B-ALL	NTPL-109	1	1	0	BCL2	5_prime_UTR_variant
B-ALL	NTPL-109	1	1	0	RUNX1	3_prime_UTR_variant
B-ALL	NTPL-109	0.9923	0.9511	-0.0412	CCND1	synonymous_variant
B-ALL	NTPL-119	0.3057	0.3492	0.0435	FGFR1	missense_variant
B-ALL	NTPL-119	1	1	0	BCL2	5_prime_UTR_variant
B-ALL	NTPL-119	0.3279	0.3024	-0.0255	RUNX1	synonymous_variant
B-ALL	NTPL-119	0.5556	0.3214	-0.2342	CHIC2	3_prime_UTR_variant
B-ALL	NTPL-127	0.335	0.8709	0.5359	NOTCH1	frameshift_variant&splice_region_variant
B-ALL	NTPL-127	0.8571	1	0.1429	ABL1	splice_donor_variant
B-ALL	NTPL-127	0.0237	0.0764	0.0527	ETV6	stop_gained
B-ALL	NTPL-127	0.9697	1	0.0303	BCL2	5_prime_UTR_variant
B-ALL	NTPL-127	0.5024	0.5025	1E-04	CCND3	missense_variant
B-ALL	NTPL-127	1	1	0	CCND2	5_prime_UTR_variant
B-ALL	NTPL-127	0.5543	0.4439	-0.1104	MYC	5_prime_UTR_variant
B-ALL	NTPL-137	0.5158	1	0.4842	CCND2	5_prime_UTR_variant
B-ALL	NTPL-137	1	1	0	BCL2	5_prime_UTR_variant
AML	NTPL-146	0.1775	0.2394	0.0619	PML	missense_variant
AML	NTPL-146	0.7665	0.8025	0.036	RAB7A	3_prime_UTR_variant
AML	NTPL-146	0.437	0.4682	0.0312	TFG	missense_variant
AML	NTPL-146	0.4177	0.4469	0.0292	BCL2	3_prime_UTR_variant
AML	NTPL-146	0.1544	0.183	0.0286	FOXP1	splice_region_variant&5_prime_UTR_variant
AML	NTPL-146	0.4374	0.4519	0.0145	CSF1R	missense_variant&splice_region_variant
AML	NTPL-146	0.0222	0.0301	0.0079	MLLT10	5_prime_UTR_variant
AML	NTPL-146	0.0605	0.067	0.0065	MLLT10	5_prime_UTR_variant
AML	NTPL-146	0.3146	0.157	-0.1576	MYC	missense_variant
AML	NTPL-146	0.6466	0.4298	-0.2168	CCND3	missense_variant
B-ALL	NTPL-155	0.1994	0.4619	0.2625	BCR	frameshift_variant&splice_region_variant
B-ALL	NTPL-155	0.0302	0.1015	0.0713	CEBPA	synonymous_variant
B-ALL	NTPL-155	0.4837	0.5487	0.065	NFKB2	synonymous_variant
B-ALL	NTPL-155	0.3898	0.4484	0.0586	NUP98	missense_variant
B-ALL	NTPL-155	0.0313	0.0801	0.0488	CEBPA	synonymous_variant
B-ALL	NTPL-155	0.494	0.5024	0.0084	NOTCH1	missense_variant
B-ALL	NTPL-155	1	1	0	CHIC2	3_prime_UTR_variant
B-ALL	NTPL-155	1	1	0	RUNX1	3_prime_UTR_variant
B-ALL	NTPL-155	0.1782	0.0862	-0.092	FOXP1	splice_region_variant&5_prime_UTR_variant
B-ALL	NTPL-155	0.8654	0.3778	-0.4876	ABL2	5_prime_UTR_variant
B-ALL	NTPL-164	0.8346	0.9859	0.1513	CCND1	synonymous_variant
B-ALL	NTPL-164	0.3125	0.4364	0.1239	CREBBP	5_prime_UTR_variant
B-ALL	NTPL-164	0.5135	0.6327	0.1192	SEMA6A	synonymous_variant
B-ALL	NTPL-164	0.8581	0.9627	0.1046	CCND1	synonymous_variant
B-ALL	NTPL-164	0.4029	0.4545	0.0516	TFG	synonymous_variant
B-ALL	NTPL-164	0.6547	0.7035	0.0488	MYC	5_prime_UTR_variant
B-ALL	NTPL-164	0.5495	0.5828	0.0333	JAK2	synonymous_variant
B-ALL	NTPL-164	0.5119	0.5358	0.0239	TCF3	synonymous_variant
B-ALL	NTPL-164	0.4528	0.4613	0.0085	CBFB	synonymous_variant
B-ALL	NTPL-164	0.4848	0.4894	0.0046	ETV6	missense_variant
B-ALL	NTPL-164	1	1	0	BCL2	5_prime_UTR_variant
B-ALL	NTPL-164	0.1625	0.1371	-0.0254	MYC	synonymous_variant
B-ALL	NTPL-164	0.0866	0.0539	-0.0327	IKZF1	missense_variant
B-ALL	NTPL-164	0.5403	0.5	-0.0403	PDCD1	synonymous_variant
B-ALL	NTPL-164	0.5608	0.4912	-0.0696	PAX5	5_prime_UTR_variant
B-ALL	NTPL-168	0.4098	0.6	0.1902	CCND1	synonymous_variant
B-ALL	NTPL-168	0.5652	0.6923	0.1271	DUSP22	missense_variant
B-ALL	NTPL-168	0.5729	0.6276	0.0547	ABL2	synonymous_variant

B-ALL	NTPL-168	0.4179	0.4571	0.0392	JAK2	synonymous_variant
B-ALL	NTPL-168	0.4837	0.5097	0.026	TCF3	missense_variant
B-ALL	NTPL-168	0.4389	0.4597	0.0208	CREBBP	missense_variant
B-ALL	NTPL-168	0.4775	0.4788	0.0013	JAK2	synonymous_variant
B-ALL	NTPL-168	1	1	0	BCL2	5_prime_UTR_variant
B-ALL	NTPL-168	1	0.9965	-0.0035	CCND2	5_prime_UTR_variant
B-ALL	NTPL-168	0.5022	0.4362	-0.066	CREBBP	synonymous_variant
B-ALL	NTPL-168	0.5	0.4297	-0.0703	IRF8	synonymous_variant
B-ALL	NTPL-20	0.4915	0.5745	0.083	PML	missense_variant
B-ALL	NTPL-20	0.6179	0.6977	0.0798	RUNX1	5_prime_UTR_variant
B-ALL	NTPL-20	0.4348	0.5057	0.0709	FOXP1	5_prime_UTR_variant
B-ALL	NTPL-20	0.98	1	0.02	CDKN2A	stop_gained
B-ALL	NTPL-20	0.4089	0.4148	0.0059	FGFR1	missense_variant
B-ALL	NTPL-20	0.4159	0.4191	0.0032	CCND3	missense_variant
B-ALL	NTPL-20	1	1	0	BCL2	5_prime_UTR_variant
B-ALL	NTPL-20	0.4685	0.4365	-0.032	TYK2	missense_variant
B-ALL	NTPL-20	0.5158	0.4706	-0.0452	PDGFRB	missense_variant
B-ALL	NTPL-216	0.9907	0.9964	0.0057	FOXP1	5_prime_UTR_variant
B-ALL	NTPL-216	0.6	0.4948	-0.1052	BCL2	5_prime_UTR_variant
B-ALL	NTPL-216	0.4293	0.3208	-0.1085	ERG	synonymous_variant
B-ALL	NTPL-26	0.6558	0.8623	0.2065	CCND1	synonymous_variant
B-ALL	NTPL-26	0.8333	1	0.1667	MLF1	5_prime_UTR_variant
B-ALL	NTPL-26	0.7081	0.8645	0.1564	CCND1	synonymous_variant
B-ALL	NTPL-26	0.022	0.0029	-0.0191	TCF3	missense_variant
B-ALL	NTPL-26	0.3308	0.1126	-0.2182	FOXP1	splice_region_variant&5_prime_UTR_variant
T-ALL	NTPL-300	0.3684	0.5272	0.1588	BCL2	5_prime_UTR_variant
T-ALL	NTPL-300	0.3797	0.5	0.1203	TYK2	missense_variant
T-ALL	NTPL-300	0.5543	0.568	0.0137	NOTCH1	missense_variant
T-ALL	NTPL-300	0.3957	0.404	0.0083	IKZF1	missense_variant
T-ALL	NTPL-300	0.8333	0.6214	-0.2119	CHD1	5_prime_UTR_variant
AML	NTPL-301	0.4775	0.5877	0.1102	ZCCHC7	5_prime_UTR_variant
AML	NTPL-301	0.3931	0.5	0.1069	TYK2	missense_variant
AML	NTPL-301	0.4889	0.5704	0.0815	BCL2	3_prime_UTR_variant
AML	NTPL-301	0.4535	0.4554	0.0019	MUC1	missense_variant
AML	NTPL-301	1	1	0	CCND2	5_prime_UTR_variant
AML	NTPL-301	1	1	0	ABL1	splice_donor_variant
AML	NTPL-301	0.5071	0.5047	-0.0024	CCND3	missense_variant
AML	NTPL-301	0.1097	0.065	-0.0447	MLLT10	5_prime_UTR_variant
B-ALL	NTPL-313	0.9375	1	0.0625	BCL2	5_prime_UTR_variant
B-ALL	NTPL-313	0.9857	0.995	0.0093	CCND2	5_prime_UTR_variant
B-ALL	NTPL-313	1	1	0	BCL2	5_prime_UTR_variant
B-ALL	NTPL-313	1	1	0	RUNX1	3_prime_UTR_variant
B-ALL	NTPL-313	0.5462	0.5087	-0.0375	PAX5	missense_variant
B-ALL	NTPL-313	0.4444	0.3983	-0.0461	NOTCH1	missense_variant
B-ALL	NTPL-313	0.5296	0.457	-0.0726	NOTCH1	synonymous_variant
B-ALL	NTPL-367	0.8481	0.985	0.1369	CCND1	synonymous_variant
B-ALL	NTPL-367	0.8614	0.9781	0.1167	CCND1	synonymous_variant
B-ALL	NTPL-367	0.3265	0.3923	0.0658	PAX5	missense_variant
B-ALL	NTPL-367	1	1	0	IKZF3	3_prime_UTR_variant
B-ALL	NTPL-367	1	1	0	BCL2	5_prime_UTR_variant
B-ALL	NTPL-367	1	1	0	BCL2	5_prime_UTR_variant
B-ALL	NTPL-367	1	1	0	RUNX1	3_prime_UTR_variant
B-ALL	NTPL-367	1	0.9994	-0.0006	CDKN2A	3_prime_UTR_variant
B-ALL	NTPL-367	0.4835	0.4704	-0.0131	PAX5	missense_variant
B-ALL	NTPL-367	0.413	0.399	-0.014	FOXP1	missense_variant

B-ALL	NTPL-367	0.5376	0.4929	-0.0447	PAX5	3_prime_UTR_variant
B-ALL	NTPL-367	0.1995	0.1464	-0.0531	FOXP1	splice_region_variant&5_prime_UTR_variant
B-ALL	NTPL-367	0.581	0.4754	-0.1056	IRF8	synonymous_variant
B-ALL	NTPL-367	0.6571	0.5161	-0.141	ABL2	5_prime_UTR_variant
AML	NTPL-377	0.4375	0.7475	0.31	CDKN2A	3_prime_UTR_variant
AML	NTPL-377	0.9355	1	0.0645	CHIC2	3_prime_UTR_variant
AML	NTPL-377	0.9918	0.9978	0.006	CCND2	5_prime_UTR_variant
AML	NTPL-377	0.4848	0.4872	0.0024	NOTCH1	3_prime_UTR_variant
AML	NTPL-377	1	1	0	BCL2	5_prime_UTR_variant
AML	NTPL-377	1	1	0	RUNX1	3_prime_UTR_variant
AML	NTPL-377	1	1	0	MECOM	5_prime_UTR_variant
AML	NTPL-377	0.5298	0.4854	-0.0444	ETV6	missense_variant
AML	NTPL-377	0.4526	0.2821	-0.1705	ABL2	5_prime_UTR_variant
T-ALL	NTPL-454	0.9778	1	0.0222	NOTCH1	3_prime_UTR_variant
T-ALL	NTPL-454	0.5652	0.5784	0.0132	RAB7A	3_prime_UTR_variant
T-ALL	NTPL-454	1	1	0	BCL2	5_prime_UTR_variant
T-ALL	NTPL-454	1	1	0	BCL2	5_prime_UTR_variant
T-ALL	NTPL-454	1	1	0	RUNX1	3_prime_UTR_variant
T-ALL	NTPL-454	0.0669	0.0493	-0.0176	MLLT10	5_prime_UTR_variant
T-ALL	NTPL-454	0.481	0.4364	-0.0446	RAB7A	3_prime_UTR_variant
T-ALL	NTPL-454	0.4124	0.3623	-0.0501	RAB7A	3_prime_UTR_variant
T-ALL	NTPL-454	0.4789	0.4286	-0.0503	RAB7A	3_prime_UTR_variant
T-ALL	NTPL-454	0.2478	0.1589	-0.0889	IKZF1	missense_variant
AML	NTPL-511	0.2381	0.604	0.3659	MYC	5_prime_UTR_variant
AML	NTPL-511	0.3171	0.4567	0.1396	TCF3	missense_variant
AML	NTPL-511	0.4528	0.3913	-0.0615	CCND3	missense_variant
T-ALL	NTPL-59	0.2111	0.8439	0.6328	CEBPA	missense_variant
T-ALL	NTPL-59	0.325	0.8889	0.5639	CEBPA	missense_variant
T-ALL	NTPL-59	0.7083	0.9262	0.2179	CEBPA	missense_variant
T-ALL	NTPL-59	0.7083	0.9262	0.2179	CEBPA	missense_variant
T-ALL	NTPL-59	0.5	0.6212	0.1212	CEBPA	frameshift_variant
T-ALL	NTPL-59	1	1	0	BCL2	5_prime_UTR_variant
T-ALL	NTPL-59	1	1	0	RUNX1	3_prime_UTR_variant
T-ALL	NTPL-59	1	1	0	CHIC2	3_prime_UTR_variant
T-ALL	NTPL-59	1	0.9857	-0.0143	CEBPA	frameshift_variant
T-ALL	NTPL-59	0.8241	0.3362	-0.4879	NOTCH1	frameshift_variant&splice_region_variant
AML	NTPL-706	0.111	0.4521	0.3411	NOTCH1	frameshift_variant&splice_region_variant
B-ALL	NTPL-796	0.3204	0.405	0.0846	MYC	5_prime_UTR_variant
B-ALL	NTPL-796	0.1773	0.2535	0.0762	FOXP1	splice_region_variant&5_prime_UTR_variant
B-ALL	NTPL-796	0.9796	1	0.0204	ABL2	5_prime_UTR_variant
B-ALL	NTPL-796	1	1	0	CCND2	5_prime_UTR_variant
B-ALL	NTPL-84	0.4134	0.4316	0.0182	CBFB	synonymous_variant
B-ALL	NTPL-84	0.5	0.5063	0.0063	NOTCH1	missense_variant
B-ALL	NTPL-84	1	1	0	BCL2	5_prime_UTR_variant
B-ALL	NTPL-84	0.0119	0.0041	-0.0078	RUNX1	5_prime_UTR_variant
B-ALL	NTPL-84	0.4653	0.4474	-0.0179	TYK2	missense_variant
B-ALL	NTPL-84	0.4333	0.3988	-0.0345	VCP	synonymous_variant
B-ALL	NTPL-84	0.7527	0.4915	-0.2612	CHD1	5_prime_UTR_variant
B-ALL	NTPL-87	0.2614	0.4071	0.1457	NOTCH1	frameshift_variant&splice_region_variant
B-ALL	NTPL-87	0.9032	1	0.0968	KAT6A	frameshift_variant&splice_region_variant
B-ALL	NTPL-87	0.0774	0.1226	0.0452	MLLT10	5_prime_UTR_variant
B-ALL	NTPL-87	1	1	0	BCL2	5_prime_UTR_variant
B-ALL	NTPL-87	1	1	0	BCL2	5_prime_UTR_variant
B-ALL	NTPL-87	0.9904	0.9893	-0.0011	TCF3	missense_variant
B-ALL	NTPL-90	0.4544	0.4957	0.0413	MLLT10	5_prime_UTR_variant

B-ALL	NTPL-90	1	1	0	BCL2	5_prime_UTR_variant
B-ALL	NTPL-90	0.3455	0.2993	-0.0462	BCL2	3_prime_UTR_variant
B-ALL	NTPL-90	0.3388	0.2568	-0.082	BCL2	3_prime_UTR_variant
B-ALL	NTPL-92	1	1	0	BCL2	5_prime_UTR_variant
B-ALL	NTPL-92	0.3068	0.1998	-0.107	PAX5	missense_variant
B-ALL	NTPL-92	0.3875	0.2455	-0.142	PAX5	missense_variant
